# It Will Take a Global Movement to Curb Corruption in Health Systems: Comment on "We Need to Talk About Corruption in Health Systems"

**DOI:** 10.15171/ijhpm.2019.58

**Published:** 2019-07-24

**Authors:** Margot I. Witvliet

**Affiliations:** Department of Sociology, Social Work and Criminal Justice, Lamar University, Beaumont, TX, USA.

**Keywords:** Corruption, Global Governance, Health Systems, Healthcare

## Abstract

Corruption in health systems is a problem around the world. Prior research consistently shows that corruption is detrimental to population health. Yet public health professionals are slow to address this complicated issue on a global scale. In the editorial entitled "We Need to Talk About Corruption in Health Systems" concern with the general lack of discourse on this topic amongst health professionals is highlighted. In this invited commentary three contributing factors that hamper public dialogue on corruption are discussed. These include (i) corrupt acts are often not illegal, (ii) government and medical professionals continued acceptance of corruption in the health systems, and (iii) lack of awareness within the general public on the extent of the problem. It is advocated that a global movement that is fully inclusive needs to occur to eradicate corruption.

The Universal Declaration of Human Rights outlines that all people have the right to security and a right to health.^1^ Yet around the world, corruption can keep a person’s right to security and right to health out of reach. Corruption is multifaceted and can manifest in many forms. Several definitions exist. The World Bank describes two types of corruption, namely (*i*) covert corruption which pertains to misdeeds carried out by those in service positions (ie, doctors, lawyers, and teachers) and (*ii*) overt corruption, which is associated to the high-profile misdeeds that usually goes viral on social media or makes the nightly news.^2-5^ Transparency International conceptualizes corruption in a global survey as ‘the abuse of public office for private gain.’^6^ In this commentary both the World Bank and Transparency International definitions of corruption are thought of when discussing the problem of corruption in health systems. It is important that the reader keep in mind that not all corruption is considered illegal. Nevertheless, corruption is particularly harmful to health systems since it hampers the ability for many to achieve adequate health care.^7^ Take for instance some of the most common forms of corruption observed in the healthcare industry, such as bribery, theft of equipment, and forms of fraud including absenteeism and inflated costs.^7-10^ For these reasons, the Sustainable Development Goals for 2030 highlight the need to significantly reduce corruption around the world.^11^

In their editorial, Eleanor Hutchinson, Dina Balabanova, and Martin McKee highlight their research agenda and plans to address corruption in health systems.^12^ One of their aims is to extend the conversation about corruption in health systems.^12^ The authors are correct to advocate that corruption in health research needs to be addressed. The United Nations (UN) highlighted this as well in the 2017 Special Rapporteur entitled “Right of everyone to the enjoyment of the highest attainable standard of physical and mental health.”^7^ The words corruption and health systems are not mentioned in the title of the UN document. However, the report is entirely concerned with how corruption impedes the right to health.^7^ Such as what is evident when clinical trials funded by industry produce biased results that support industry, as opposed to results produced by clinical trials with no industry funding.^7^ In this case industry profit is more of a concern then the right to health. The UN Special Rapporteur provides recommendations, most notably being the need to raise awareness amongst the actors working in the health system and in the general population.^7^ The UN also notes that it is advantageous to use the right to health framework since it provides a legal manner to address corruption in health systems.^7^ If we are going to amplify the conversation on corruption in health systems, then research agendas should include the recommendations made by the UN. Corruption is a global problem that affects all sectors of society in both high- and low-income countries. In this invited commentary three contributing factors impeding the amplified public dialogue on corruption are discussed.

First, many corrupt acts are not illegal. In the US laws fuel corruption in the health system. This form of corruption is closest to the conceptualization of corruption by Transparency International. For example, it is entirely legal for an American policy-maker to accept funds from corporate political action committees (eg, healthcare or the pharmaceutical industries), and then go on to shape healthcare policy.^13^ In the United States, corporate backed policy-makers are not limited in the number of bills they can introduce or support that will later become laws.^13^ No laws exist in the United States that prevents the policy-maker or their family members and acquaintances from holding stocks in the corporations that funds the policy-maker’s time in political office.^13^ As such, the policy-maker is theoretically free to shape health policy that impacts health systems. This corrupt act is not illegal, yet it might influence the stock market and thereby provide financial rewards to the policy-maker or their family members and acquaintances that have stocks in the health market.^13^ Canada on the other end of the spectrum is a country that recently took a step to curb the influence big corporations have on policy-makers. Canadians implemented a new law that bans corporations from donating large sums of money to policy-makers.^14^ The aim for this change in law is to reduce the influence corporations might have on shaping policy, including health policy.

Policy-makers and decision-makers who have influence on the health system are often driven by self-interests. This makes it incredibly difficult to shape laws and regulations that effectively combat corruption in health systems. Even when policy-makers and decision-makers are not corrupt, research shows that they might choose to implement anti-corruption measurements that will not fully eradicate corruption when it applies to their own work.^15^ This means that those who historically advocate for curbing corruption are not immune to rigging the system. Prior research finds that decision-makers might implement weaker anti-corruption measures.^15^ This perhaps explains why even in countries where ethics committees are prevalent and anti-corrupt taskforces are in place, corruption is still rampant and in many cases the corrupt acts are legal. Research suggests that if the decision-maker receives immunity from punishment, then this can motivate them to develop stricter anti-corruption policies.^15^ Future corruption and health research will benefit from taking this phenomenon into account. In addition, conducting natural experiments on countries like Canada to examine the effects of recent changes in how the political system is financed will be advantageous to the literature.

The second factor that needs consideration is that corrupt acts are often not recognized as being corrupt, especially when government and medical professionals condone the act. In some nations bribery is part of the fabric of society, especially in countries within Africa.^16^ In India, as in many other low-income countries, bribery payments to doctors is a huge problem.^17^ Cases in India have been reported where women are forced to make a bribery payment to hold their babies after delivery.^17^ Around the world costs of labor and delivery should be transparent and costs for deliveries without complications uniform. However, for many countries this is not the case. In the United States, hospitals are allowed to overcharge for pharmaceuticals.^18^ The price of headache medicine in a US store costs less than $2.00, but in a hospital a single over-the-counter headache pill can cost over $10.^18^ Hospital medical equipment and maintenance of the equipment is also expensive.^18^ This form of corruption is allowed by government in the United States and it is considered normative practice by medical professionals. Americans view this corrupt act as normal because government has not stepped up to create laws against such practices. Government has made price gouging in stores in most US states illegal^19^ and it should also be made illegal in health systems. In addition, researchers have even identified a link between increased payments of foreign aid and UN security-council membership.^20^

Third, in discussions on combating corruption in health systems increasing awareness about corruption in health systems and the subsequent harm caused by corrupt acts in the general public is key.^7^ Most people are busy trying to sustain themselves and their families economically. The public has not become outraged enough about corruption in health systems to collectively advocate for change. Most people are not aware that research shows corruption negatively affects child health outcomes and disproportionately harms the health of vulnerable groups.^21^ Few people in the general public know that women and poor people are typically the victims of corruption in health systems and that this has a major impact on their health.^22^ Although it is evident that in societies where corruption is rampant, everyone’s health suffers,^23^ government officials continue to allow corruption to have a place in society even at the sake of jeopardizing population health.

All nations around the world share the commonality of corruption in health systems. A global anti-corruption movement must come with collective action. The authors of the editorial “We need to Talk About Corruption in Health Systems” are ready to ignite a collective conversation on corruption and health systems, but is the world and in particular is government ready? The #MeToo movement was originally started by an African American woman over a decade ago, but the #MeToo movement has only recently gained global awareness.^24^ What is clear is that this generation of health professionals are on the cusp of making a major change to health systems. Internet has made us a global village. As such, we are not able to say we are not aware of our social problems. Globalization is not a new phenomenon, but with the advent of modern technology it has transformed the world into a relatively smaller place. Media has put a face to corruption occurring around the world. Given that upcoming health professionals are highly informed about global problems, and interests in health systems are increasing, the elements for a successful global movement are in place.

Raising societal awareness about corruption in health systems is critical because the healthcare industry is one of the most corrupt industries in the world.^25^ Any steps taken to eradicate corruption will need to account for the huge amount of diversity both between and within countries. This requires not only a multi-disciplinary approach, but also a fully inclusive approach that encompasses different perspectives from health researchers in both low- and high-income countries.

## Ethical issues

Not applicable.

## Competing interests

Author declares that she has no competing interests.

## Author’s contribution

MIW is the single author of the paper.

## References

[1] United Nations. Universal Declaration of Human Rights. https://www.un.org/en/universal-declaration-human-rights/. Accessed April 2, 2019. Published 1948.

[2] The World Bank. Africa Development Indicators 2010. http://www-wds.worldbank.org/external/default/WDSContentServer/IW3P/IB/2010/04/07/000333037_20100407014412/Rendered/PDF/538800PUB0AFR0101Official0Use0Only1.pdf. Accessed April 3, 2019. Published 2010.

[3] National Public Radio. https://www.npr.org/tags/522920705/police-shootings. Accessed April 15, 2019. Published 2017.

[4] Joe Tacopino J. FEMA head reportedly spent $150K on unauthorized travel. NYPost. September 26, 2018. https://nypost.com/2018/09/26/fema-head-reportedly-spent-150k-on-unauthorized-travel/. Accessed March 25, 2019.

[5] National Public Radio. https://www.npr.org/2018/02/28/589493664/ben-carson-and-hud-face-allegations-of-lavish-spending-including-31-000-furnitur. Accessed March 25, 2019. Published 2018.

[6] Transparency International. Global Corruption Report 2006. Ann Arbor, MI: Pluto Press; 2006.

[7] United Nations. Right of everyone to the enjoyment of the highest attainable standard of physical and mental health. New York: United Nations; 2017.

[8] Vian T (2008). Review of corruption in the health sector: theory, methods and interventions. Health Policy Plan.

[9] Lewis M. Governance and Corruption in Public Health Care Systems. Center for Global Development Working Paper No.78. http://papers.ssrn.com/sol3/papers.cfm?abstract_id=984046. Accessed March 29, 2019. Published 2006.

[10] Savedoff W, Hussmann, K. Why are health systems prone to corruption? In: J Kotalik J, D Rodriguez D, eds.Global Corruption Report 2006. Arbor, MI: Pluto Press, Ann; 2006:3-13.

[11] United Nations. Sustainable Development Goals 2030. https://sustainabledevelopment.un.org/?menu=1300. Accessed April 25, 2019. Published 2015.

[12] Hutchinson E, Balabanova D, McKee M (2019). We need to talk about corruption in health systems. Int J Health Policy Manag.

[13] Business Insider. Alexandria Ocasio-Cortez invented a ‘Corruption Game’ to slam lax government ethics laws during a viral Oversight Committee hearing. https://www.businessinsider.com/alexandria-ocasio-cortez-slams-corruption-in-oversight-hearing-2019-2. Accessed April 1, 2019. Published 2019.

[14] CBC. Companies still made largest political donations, ahead of ban. https://www.cbc.ca/news/canada/new-brunswick/new-brunswick-political-donations-ban-companies-2017-1.4457205. Accessed April 9, 2019. Published 2017.

[15] Boly A, Gillanders R (2018). Anti-corruption policymaking, discretionary power and institutional quality: An experimental analysis. J Econ Behav Organ.

[16] Peiffer C, Rose R (2016). Why Are the Poor More Vulnerable to Bribery in Africa? The Institutional Effects of Services. J Dev Stud.

[17] Jain A, Nundy S, Abbasi K (2014). Corruption: medicine’s dirty open secret Doctors must fight back against kickbacks. BMJ.

[18] Rosenthal E. An American Sickness: How Health Care Became Big Business and How You Can Take It Back. New York, NY: Penguin Press; 2017.

[19] Harvard Business Review. The problem with price gouging laws. https://hbr.org/2013/07/the-problem-with-price-gouging-laws. Assessed March 3, 2019. Published 2013.

[20] Kuziemko I, Werker E (2006). How much is a seat on the security council worth? Foreign aid and bribery at the United Nations. Journal of Political Economy.

[21] Azfar O, Gurgur T (2008). Does corruption affect health outcomes in the Philippines?. Economics of Governance.

[22] Hossain N, Nyamu-Musembi C. Corruption accountability and gender: understanding the connections. http://www.womenpoliticalparticipation.org/upload/file/UNDP-UNIFEM_Corruption,%20Accountability%20and%20Gender_EN.pdf. Accessed March 19, 2019. Published 2010.

[23] Witvliet M, Kunst A, Arah OA, Stronks K (2013). Sick regimes and sick people: a multilevel investigation of the population health consequences of perceived national corruption. Trop Med Int Health.

[24] MeToo website. https://metoomvmt.org/about/. Assessed April 27, 2019. Published 2006.

[25] United Nations. Healthcare among most corrupt sectors, warns UN expert, backing “citizen whistleblowers.” https://www.ohchr.org/EN/NewsEvents/Pages/DisplayNews.aspx?NewsID=22283&LangID=E. Accessed April 3, 2019. Published 2017.

